# Effect of melatonin on postoperative cognitive function in elderly patients submitted to transurethral resection of the prostate under spinal anesthesia^☆^

**DOI:** 10.1016/j.clinsp.2024.100562

**Published:** 2024-12-26

**Authors:** Cristiane Tavares, Cláudia Maia Memória, Luiz Guilherme Villares da Costa, Vinícius Caldeira Quintão, Alberto Azoubel Antunes, Deborah Teodoro, Maria José Carvalho Carmona

**Affiliations:** a Hospital das Clínicas, Faculdade de Medicina, Universidade de São Paulo (HCFMUSP), São Paulo, SP, Brazil; b Hospital Israelita Albert Einstein, São Paulo, SP, Brazil

**Keywords:** Cognitive Dysfunction, Melatonin, Nocturia, Perioperative Care, Postoperative Cognitive Complications, Transurethral Resection of Prostate

## Abstract

**Background:**

Hospitalization for Transurethral Prostate Resection (TURP) involves circadian rhythm disturbance – a possible cause of Postoperative Neurocognitive Disorder (POCD) in elderly patients. This study investigated whether melatonin ameliorated this effect.

**Methods:**

A double-blind, randomized clinical trial used a battery of neuropsychological tests to evaluate cognitive performance of 118 patients aged ≥ 60, before TURP with spinal anesthesia, and at 21- and 180-days PO. Patients received 10 mg of melatonin, or a placebo, on the night before surgery and 1-, 2- and 3-days PO. Delayed neurocognitive recovery in the two groups at 21 days PO was compared using the Chi-Squared test; individual performances in each test at each date were compared using the General Mixed Model. Results with p < 0.05 were considered significant.

**Results:**

Pre-surgery, both groups had significant cognitive deficits. Delayed cognitive recovery at 21 days PO was the same in both. There were no cases of POCD at 180 days. The melatonin group performed better in the delayed-recall FOME, which assesses memory, and in the Digit Span test, which assesses attention and cognitive flexibility. Unexpectedly, global neurocognitive performance was improved at 180 PO in both groups.

**Conclusions:**

Melatonin had no statistical effect on POCD, but a selective beneficial effect was observed in two cognitive areas. The high prevalence of preoperative cognitive impairment may be related to the lower urinary tract symptoms which were reasons for the surgery; the unexpected improvement of cognitive performance in all patients at 180 days PO may reflect alleviation of these symptoms.

## HIGHLIGHTS

• Perioperative Neurocognitive Disorders (PND) can occur in patients submitted to elective surgical procedures under general or regional anesthesia, especially the elderly.
• Circadian rhythm disruptions have harmful effects on cognition and may also play a role in the development of PND.
• Melatonin 10 mg administered in the perioperative period of TURP surgery for elderly patients hasn't shown any protective effect against postoperative cognitive decline, mainly because the patients have shown a significant improvement in cognitive functions after the surgery.

## Introduction

Perioperative Neurocognitive Disorders (PND) can occur in patients submitted to elective surgical procedures under general or regional anesthesia, especially the elderly. The physiopathogenic mechanisms are not yet completely known. Neuroinflammation appears to play a predominant role, as also do microglial activation and oxidative stress.^2^^,^^3^

Circadian rhythm disruptions have harmful effects on cognition and may also play a role in the development of PND.^4^ Melatonin, secreted by the pineal gland during sleep, is one of the main signalers of the circadian rhythm^5^ and one of the most powerful natural antioxidants.^6^ Its secretion decreases in older people, and also during the perioperative period,^7^ so it has a significant function in this context. Exogenous melatonin was given as tablets have well-documented anxiolytic effects before surgery, with negligible side effects,^8^ but few studies have addressed its use for the prevention of PND.

TURP is one of the most common elective surgeries performed in older men. It is practically a standardized procedure, usually performed under spinal anesthesia, with very low mortality and morbidity rates.^9^ Hospitalization, with its changes in routine, can cause circadian rhythm disturbances in elderly patients and may have a preponderant role in the development of PND in a population undergoing this surgery. The present hypothesis was that melatonin might alleviate this disturbance of circadian rhythm; and our choice of a single type of surgery and anesthesia (spinal anesthesia) would serve to exclude other factors such as general anesthetic agents, significant bleeding, intensity of postoperative pain, etc., which are not present in this surgery/anesthetic combination.

The present study aimed to evaluate the perioperative cognitive function of elderly patients undergoing TURP with spinal anesthesia, and the effect of administration of exogenous melatonin, in comparison to a placebo, on the prevalence of delay in cognitive recovery in these patients.

## Methods

The authors carried out a double-blind, parallel, randomized placebo-controlled clinical trial in patients aged ≥ 60 undergoing elective TURP under spinal anesthesia at Instituto Central of Hospital das Clínicas HCFMUSP, Faculdade de Medicina, Universidade de São Paulo, São Paulo, SP, BR. It was registered on the research platform of Brazil's National Health Council, and on the public website www.clinicaltrials.gov under the identifier NCT03966950.

The primary outcome was to compare the prevalence of Postoperative Neurocognitive Disturbances (PND) in the group that received melatonin and the placebo group, at 21 days PO (when they are still considered as a delay in neurocognitive recovery)[1] and at 180 days PO.

The secondary outcome of the study was to evaluate the cognitive performance of the patients in each test on the three dates (pre-operative, 21 days PO, and 180 days PO).

Institutional Human Research Ethics Committee approval (Supplemental Digital Content 1) was granted, and written informed consent was obtained from all patients (Supplemental Digital Content 2).

The intervention took the form of oral doses of 10 mg of melatonin (Natrol Laboratory, USA), given on the night before the surgery and on the first three nights of the postoperative period. The tablets of melatonin and the placebo were presented in identical packaging and prescribed in the same way. The randomization was carried out in blocks of ten, using the software randomization.com. A pharmacist of the Pharmacy Division of the Central Institute of Hospital das Clínicas was responsible for labeling the packages, containing three tablets of melatonin or three tablets of placebo each, sequentially numbered, and for keeping the randomization identification list concealed until the end of the study. The allocation ratio was 1:1.

Eligible patients were males aged over 60 with Benign Prostate Hyperplasia (BPH), scheduled for elective prostrate transurethral resection surgery, under spinal anesthesia, who obtained a minimum Mini-Mental State Examination (MMSE) score of 18 (for those with more than one year's schooling) or 23 (for those with more than four years’ schooling).^10^

Exclusion criteria were psychiatric or neurological diseases such as schizophrenia, Parkinson's Syndrome, epilepsy, severe traumatic brain injury, prior neurosurgery, cancer, or significant hearing or visual impairment even with glasses or a hearing aid, patients that were illiterate or did not understand Portuguese, or who refused to take part in the study, or ceased at any time.

All participants, investigators, care providers, and statisticians were blinded. Care providers included surgeons, anesthesiologists, neuropsychologists, and the nursing team.

The battery of neuropsychological tests, with a duration of 60–80 minutes, comprised: the Digit-Symbol coding; a Digit Span test; the Trail Making Test, parts A and B; the Stroop Color and Word Test; the Fuld Object Memory Evaluation test (FOME); the delayed recall FOME test; the Rey Complex Figure Test for copy and 30-minute recall; and semantic fluency tests on names of animals and fruits. This group of tests was applied in a quiet, illuminated environment, on three dates: before surgery, and 21- and 180-day PO. Different versions of the tests were used on different dates, when they existed, to avoid any ‘learning-effect’ distortions.^11^

For the sample calculation, the authors assumed a prevalence of 10% for the occurrence of delay in neurocognitive recovery^12^ and that we would regard a reduction from 10% to 3% in the group receiving the intervention as clinically significant. With significance set at 0.05 and power set at 80% plus an estimated dropout rate of 10%, the sample size required for that reduction was 426 patients (213 in each group).

Since there were no prior studies on the prevalence of delay in cognitive recovery, specifically in this population, an interim analysis was planned after 30% of the sample completion, using the O'Brien-Fleming approach.^13^

Group comparisons were made using unpaired *t*-tests or the Mann-Whitney test for continuous variables, and the χ^2^ or Fisher's exact test for dichotomous variables. The Type I rate was controlled using the Holm-Bonferroni step-down procedure for multiple comparisons.^14^, ^15^, ^16^

For the primary outcomes, the authors used the χ^2^ test. Changes in neuropsychological tests were compared, using *z*-score analysis, with the results of Standard Tables for the Brazilian population matched for age, gender, and level of schooling, reported as years of formal education.^16^, ^17^, ^18^

Using Rasmussen et al., 2004, as a point of departure,^11^ delay in neurocognitive recovery (in an individual patient) was defined as: (a) If the *z*-score of a patient in any two (or more) of the 11 tests declined by 1.96 or more; or (b) If that patient's composite *z*-score (for the 11 tests as a whole) declined by 1.96 or more at 21 days PO in comparison with the pre-operative score. Postoperative neurocognitive disturbance was defined accordingly,^1^ comparing 180 days PO with the pre-operative score.

For the secondary outcome, the authors used the General Mixed Model (GMM), to capture fixed as well as random effects and slopes. The authors used the *z*-score of each patient in each test and also the composite *z*-score to evaluate the global neurocognitive performance. The composite *z*-score was calculated as the arithmetic mean of the individual values for each of the 11 tests applied.

Missing data were considered MAR (Missing at Random) and were planned to be handled by adopting multiple imputations, using the Last Observation Carried Forward method (LOCF).

Statistical analysis was performed using version 4.1.1 of the software R (https://www.r-project.org). All the tests were carried out for a significance level of 5%. Exact binomial confidence intervals were calculated using the Clopper-Pearson interval.

## Results

Screening of 288 patients resulted in 176 patients for randomization. From those, 28 patients who received only the first doses of melatonin and 29 who received placebo were excluded from the analysis since they did not undertake the TURP procedure under spinal anesthesia for reasons unrelated to the clinical trial intervention.

**Figure 1:**
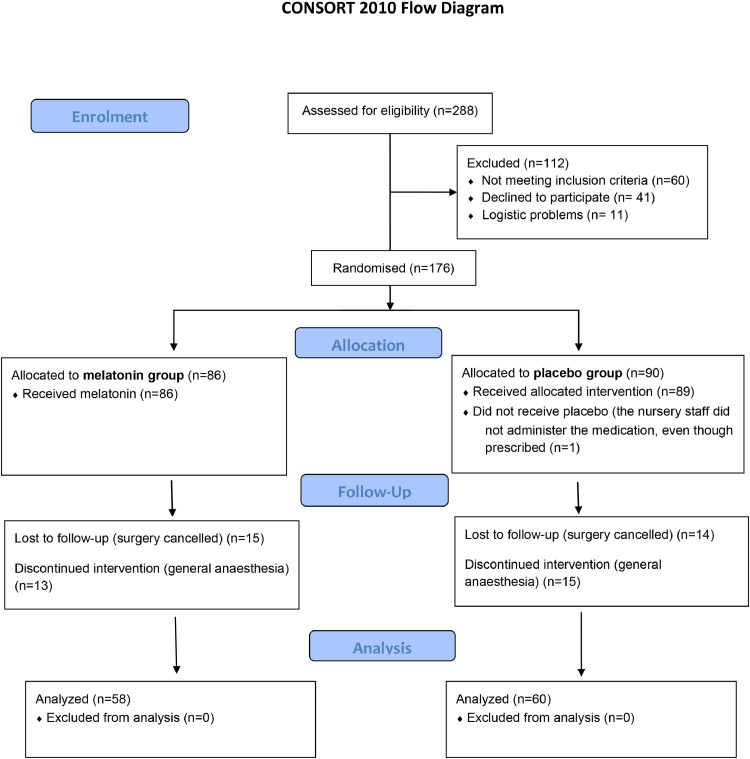
Flow Diagram of the study.

Fig. 1 is a flow diagram of the study, following the CONSORT recommendations:[18] Data were collected for 118 patients and compiled on the REDCap (Research Electronic Data Capture) platform. The total percentage of missing data was 18.2% and was handled as planned in the project design.

The interim analysis was performed and showed no statistically significant difference between the two groups. A futility analysis was undertaken using a transformed *Z*-test value (B-value) to calculate conditional power, which was 0.01%, corresponding to a futility of 99.99%. Therefore, the authors decided to terminate the study for futility.^15^

Table 1 shows the characteristics of the patients and perioperative risk factors for PND in each group.

**Table 1:** Characteristics of patients and perioperative risk factors for PND in each group.

Patient characteristics	Melatonin group (n = 58)	Placebo group (n = 60)	p
Age (years), median (IQI)	70 (66‒76)	68 (65‒75)	0.226
Years of schooling, median (IQI)	5.5 (4‒11)	4 (3‒8)	0.258
Continue to work, n (%)	11 (9.3)	9 (7.6)	0.742
MMSE, median (IQI)	26 (24‒28)	26 (23‒28)	0.537
BMI (Kg/m^2^), mean (SD)	26.33 (3.65)	25.98 (4.05)	0.627
Hypertension, n (%)	34 (28.8)	31 (26.3)	0.566
Diabetes mellitus, n (%)	9 (7.6)	11 (9.3)	0.871
Chronic renal disease, n (%)	6 (5.0)	3 (2.5)	0.455
Hypothyroidism, n (%)	2 (1.7)	2 (1.7)	0.635
Smoking, n (%)	35 (29.7)	40 (33.9)	0.602
ASA 1, n (%)	3 (2.5)	3 (2.5)	0.707
ASA 2, n (%)	47 (39.8)	50 (442.4)	0.932
ASA 3, n (%)	8 (6.8)	7 (5.9)	0.944
Functional capacity (METs)			
< 4, n (%)	8 (6.8)	10 (8.5)	0.859
4–6, n (%)	45 (38.1)	45 (38.1)	0.909
≥ 7, n (%)	5 (4.2)	4 (3.4)	0.958
Coronary disease, n (%)	7 (5.9)	5 (4.2)	0.714
Cardiac arrhythmia, n (%)	3 (2.5)	3 (2.5)	0.707
POCD, n (%)	5 (4.2)	4 (3.4)	0.958
Use of glasses, n (%)	2 (1.7)	1 (0.8)	0.539
Auditory impairment needing hearing aid, n (%)	1 (0.8)	1 (0.8)	0.981
History of delirium, n (%)	0	1 (0.8)	0.243
Preoperative pain complaint, n (%)	24 (20.3)	24 (20.3)	0.972
History of depression, n (%)	1 (0.8)	0	0.232
Severe preoperative cognitive impairment, n (%)	25 (43.1)	23 (38.3)	0.234
Moderate preoperative cognitive impairment, n (%)	7 (12)	10 (16.7)	0.876
Mild preoperative cognitive impairment, n (%)	2 (3.4)	8 (13.3)	0.325
Use of midazolam intraoperatively, n (%)	41 (34.7)	40 (33.9)	0.638
Intravesical catheter preoperatively, n (%)	22 (18.6)	16 (13.5)	0.266

IQI, Interquartile interval; MMSE, Mini-Mental State Examination; BMI, Body Mass Index; ASA, American Society of Anesthesiologists; MET, Metabolic Equivalent of Task; POCD, Postoperative Neurocognitive Disorder.

The definitions adopted for preoperative cognitive deficit were based on Ladeira et al.:[16]

Severe: Patient has two or more tests with *z*-score of -1.96, or greater negative value.

Moderate: Patient has two or more tests with *z*-score of -1.5, or greater negative value.

Mild: Patient has two or more tests of the same cognitive domain with z-score of -1 (or greater).

The authors found a statistically significant improvement in depression symptoms, quality of sleep and subjective perception of quality of life in both groups, as shown in Table 2.

**Table 2:** Association tests for anxiety and depression scores, subjective perception of quality of life, and Pittsburgh Sleep Quality Index, for each group and at each time (preoperatively and 180-days PO).

	Melatonin group			Placebo group		
	Pre-operative	180 PO	p	Pre-operative	180 PO	p
**Anxiety score: median (IQI 25‒75)**	6 (3‒8)	5 (3‒7)	<0.1^a^	6 (3.75‒7.25)	5 (3‒7)	0.05^a^
**Depression score: median (IQI 25‒75)**	3 (2‒8)	2 (1‒4)	<0.01^a^	4 (2‒8)	2 (1‒6)	0.03^a^
**Pittsburgh sleep quality index: median (IQI 25‒75)**	6 (4‒8)	4 (3‒6)	<0.01^a^	6 (4‒9)	4.5 (3‒7)	<0.001^a^
**Subjective quality of life evaluation: n (%)**						
Excellent	1 (1.72%)	8 (13.8%)	<0.001^b^	2 (3.3%)	5 (8.3%)	<0.001^b^
Very good	7 (12.06%)	8 (13.8%)	<0.001^b^	9 (15%)	11 (18.3%)	<0.001^b^
Good	34 (58.6%)	30 (51.7%)	0.5^b^	32 (53.3%)	34 (56.6%)	0.52^b^
Fair	13 (22.4%)	6 (10.3%)	<0.001^b^	16 (26.6%)	10 (16.6%)	<0.001^b^
Poor	2 (3.4%)	0	<0.001^b^	1 (1.66%)	0	<0.001^b^

a Wilcoxon signed-rank test.

b McNemar test with correction for continuity.

The total prevalence of delay in neurocognitive recovery was 10.2% (95% CI [5.37; 17.09], p = 0.113), comprising 15.5% (95% CI [7.35; 24.42] p = 0.113) in the group that received melatonin and 5% (95% CI [1.04; 13.92], p = 0.113) in the placebo group. As shown in Table 3, when the χ^2^ test was applied with continuity correction, this difference was not considered to be statistically significant (p = 0.113). None of the patients had a decline in neurocognitive functions at 180 days PO, compared with the pre-operative evaluation.

**Table 3:** Prevalence of delay in neurocognitive recovery, by group.

Variable	Category	Cognitive deficit at 21-days PO		p-value^a^
		No (n = 106)	Yes (n = 12)	
Group	Melatonin	49 (46.2%)	9 (75%)	0.113
	Placebo	57 (53.8%)	3 (25%)	

a χ^2^ test with continuity correction.

**Figure 2:**
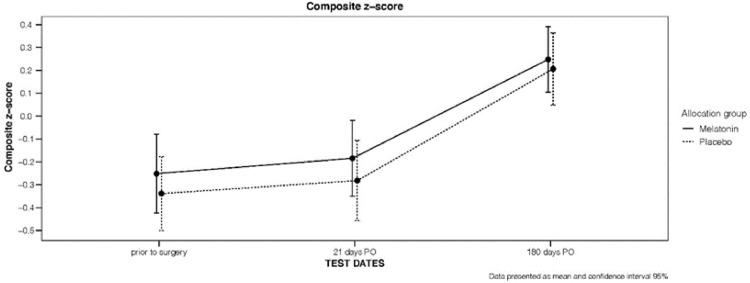
Composite *z*-score: Range and average in each group on the 3 test dates.

In relation to the secondary outcomes, longitudinal variations of the composite z-score for each group are shown in Fig. 2.

The main findings of the GMM for the z-scores of each test and for composite z-scores are summarized in Table 4.

**Table 4:** GMM for the *z*-scores, considering group and time effects.

Test	Group effect	Time effect
FOME (Fuld Object Memory Evaluation)	None	At 21-days PO, decline, -1.18 (95% CI [-0.32; -0.03], p = 0.018] from preoperative evaluation
FOMEr (Delayed-recall Fuld Object memory Evaluation)	*z*-score in the placebo group was lower -0.24 (95% CI [-0.47; -0.01, p = 0.044) than in the melatonin group	At 21-days PO, decline, -0.2 (95% CI [-0.37; 0.03], p = 0.023] from preoperative evaluation
		At 180-days PO, improvement, 1.83 (95% CI [1.66; 2.00] p < 0.001) from preoperative evaluation
Delayed-recall Rey Complex Figure Test	None	At 21-days PO, improvement, 0.24 (95% CI [0.09; 0.38], p = 0.002) from preoperative evaluation
Trail Making Test – Part A	None	At 21-days PO, improvement, 0.32 (95% CI [0.13; 0.51], p = 0.001) from preoperative evaluation
		At 180-days PO, improvement, 0.35 (95% CI [0.16; 0.54], p < 0.001) from preoperative evaluation
Digit Span Test	*z*-score in the melatonin group higher, by 0.28 (95% CI [0.01; 0.55], p = 0.046) than in the placebo group	None
Stroop	None	At 21-days PO, improvement, 0.32 (95% CI [0.08; 0.56], p = 0.009) from preoperative evaluation
Trail Making Test – Part B	None	At 21-days PO, improvement, 0.25 (95% CI [0.05; 0.45], p = 0.013) from preoperative evaluation
Digit Symbol Test	None	None
Rey Complex Figure Test	None	None
Semantic Fluency – fruits	None	At 180 PO, improvement, 1.27 (95% CI [1.07; 1.47] p < 0.001) from preoperative evaluation
Semantic Fluency – animals	None	At 180-days PO, improvement, 1.9 (95% CI [1.7; 2.1] p < 0.001) from preoperative evaluation
Composite *z*-score	None	At 180-days PO, improvement, 0.52 (95% CI [0.46; 0.59] p < 0.001), from preoperative evaluation

95% CI, Confidence Interval 95%; p, p-value.

## Discussion

In this randomized study, the authors found that administration of melatonin over four days, on the night before surgery and on the three nights after surgery, had no statistical effect on the prevalence of delayed neurocognitive recovery after 21 days of the surgery, compared to placebo.

There are heterogeneous definitions of delayed neurocognitive recovery, previously called early POCD, which limits comparability between studies. They commonly differ in the assessment dates and kinds of neuropsychological tests applied.^19^ However, it is possible that some cognitive domains are more vulnerable than others to the deleterious effects of anesthesia and surgery.^20^ The authors believe, that to capture the selective impairments in other studies in the future, it would be necessary to analyze different kinds of neuropsychological tests separately.

The authors observed a selective beneficial effect of melatonin in delayed-recall Fuld Object Memory Evaluation, which assesses memory,^21^ and in the Digit Span Test, which assesses attention and cognitive flexibility.^22^ Though this is an exploratory finding, it could indicate a possible neuroprotective effect specifically in these cognitive functions.

Recent evidence of a neuroprotective effect of melatonin was seen in preclinical studies in animals at doses higher than those used in this study. In mice, 4 mg/kg given intravenously 30 minutes before a programmed focal cerebral ischemic lesion minimized the consequences of the event.^23^ Neuroprotective effects of melatonin include anti-apoptotic and anti-inflammatory effects, and also modulation of cellular enzymes involved in cellular protection against oxidative damage.^24^

Administered orally, melatonin is also able to cross the blood-brain barrier; the dose in this study, however, was probably insufficient to produce a robust neuroprotective effect.^24^

Contrary to the authors’ expectation, all the patients submitted to transurethral prostate resection under spinal anesthesia enjoyed cognitive improvement after 180 days of the surgery, independently of the use of melatonin.

The authors considered that this might be due to improvement in the patients’ sleep quality after surgery, which we measured by the Pittsburgh sleep quality index pre- and postoperatively.

Patients with benign prostate hyperplasia frequently complain of nocturia and other obstructive symptoms of the lower urinary tract that adversely affect sleep quality. There are indications that transurethral prostate resection can reverse, or at least attenuate, these lower genital-urinary obstructive symptoms, resulting in an improvement, albeit partial, in sleep quality, without the inconvenience of the drugs used in standard (non-surgical) treatment.^25^

This proposed biological reasoning may also be corroborated by the surprisingly high finding of moderate or severe cognitive deficits in patients at the pre-surgery stage. Based on only the psychometric criteria of the pre-operative evaluation, approximately 55% of the patients would have been diagnosed with a moderate or severe cognitive deficit, and 8.5% with mild cognitive disorder. The great majority of them, however, did not present spontaneous cognitive complaints: this leads us to suspect that the symptoms of their prostate condition troubled them much more. Hence, low sleep quality arising from obstructive symptoms of the lower urinary tract may be responsible for low performance in the neurocognitive tests before surgery.

As to the limitations of this study, there are two main factors: (i) The choice of 21 days for the first follow-up tests may not have been optimal; and (ii) The complexity of differentiations in the current definitions of POCD.

The period of 21 days PO for the first neuropsychological assessment was chosen because this would coincide with the patients’ scheduled return visits, but if the tests had been carried out a few days later certain factors might have been absent (e.g., some patients had retained the catheter since surgery, and some had not).

If the first follow-up tests had been performed later, the prevalence of delayed neurocognitive recovery might have been lower (for example, because some of the procedures carried out at the return visits may have improved the patient's clinical condition).

The definitions of POCD adopted in the design of the study were those in use at the time, but these are currently being questioned. A decline in neurocognitive functions with aging is expected, with or without any anesthetics or surgery. So there is the possibility that an observed POCD is merely a function of normal fluctuation of a patient's neurocognitive functions, or simply a result of the patient's neurocognitive trajectory.^26^

Recent studies have indicated weaknesses in the definitions that were adopted in this study and the need for a nomenclature better adapted to the different vulnerabilities of patients and new ways of assessing them.^27^

There is also the possibility of using computerized tests, which can take less time and provide even more complex data.^28^

The COVID-19 pandemic resulted in another limitation of this study, due to some patients being prevented from undergoing the final tests at 180 days PO. Evaluation by telephone interviews for cognitive status had limitations. Loss of follow-up by some patients was expected, but as provided for in the study plan, its effect was mitigated by using intention-to-treat analysis with multiple imputations of data using conservative approaches.

There can be no doubt that POCD has many causes – including surgical-anesthetic stress response, bleeding, pain, use of opioids, changes in circadian rhythm, and indeed necessary hospital procedures. One strength of this present study is that, due to having chosen one single type of surgery to research, with one particular type of anesthesia (spinal anesthesia), it has shown a case of the surgery itself apparently causing an improvement in neurocognitive ability.

There are two other strong points. The first is that, in contrast to most previous studies, the authors used a very complete battery of neuropsychological tests. The second is that the authors compared the resulting *z*-scores for each test/patient with the normative data for the Brazilian population – likely producing a more faithful reflection of local cultural factors than if, for example, we used a normative data set for another country.

In future studies, standardization of the nomenclature and criteria for defining POCD, as proposed by Evered et al.,^1^ offers the possibility of more effective comparisons between the results of interventions aimed to prevent postoperative cognitive decline. Specific assessment of different cognitive domains could also facilitate the development of neuroprotective strategies that act specifically on those neurocognitive functions in which the patient is more vulnerable.

The exploratory findings of this study suggest a specific neuroprotective effect of melatonin on neurocognitive functions related to memory, attention and cognitive flexibility. These functions are compromised early in patients with Alzheimer's disease.^29^ Testing of melatonin on patients with mild cognitive impairment (amnestic MCI) who will undergo elective anesthesia or surgery may be promising or clinically significant.

It is suggested that in future studies higher doses of melatonin than those used in this study, administered for a longer period of time, could be tested.

The conclusion from the study is that in a population of elderly patients undergoing TURP with spinal anesthesia, with high preoperative prevalence of cognitive impairment, oral administration of 10 mg of melatonin on the day before surgery and on the first three nights PO had no effect on prevalence of delay in neurocognitive recovery; but may have caused better performance in tests that assessed memory, attention and cognitive flexibility. Also of interest is the observation that all patients in the study had improved cognitive ability after 180 days – which the authors suggest might be associated with the patients’ relief from their symptoms, which had been alleviated by the surgery.

## Funding

The work was supported by: Hospital das Clínicas HCFMUSP, Faculdade de Medicina, Universidade de São Paulo, São Paulo, Brazil.

## Declaration of competing interest

The authors declare no conflicts of interest.

## Appendix Supplementary materials

Image, application 1

Image, application 2

Image, application 3
