# Supplementary figures and images for: Effect of melatonin on postoperative cognitive function in elderly patients submitted to transurethral resection of the prostate under spinal anesthesia
Source: Clinics (Sao Paulo). 2024 Dec 26;80:100562. doi: 10.1016/j.clinsp.2024.100562 (PMC11732585; doi:10.1016/j.clinsp.2024.100562)

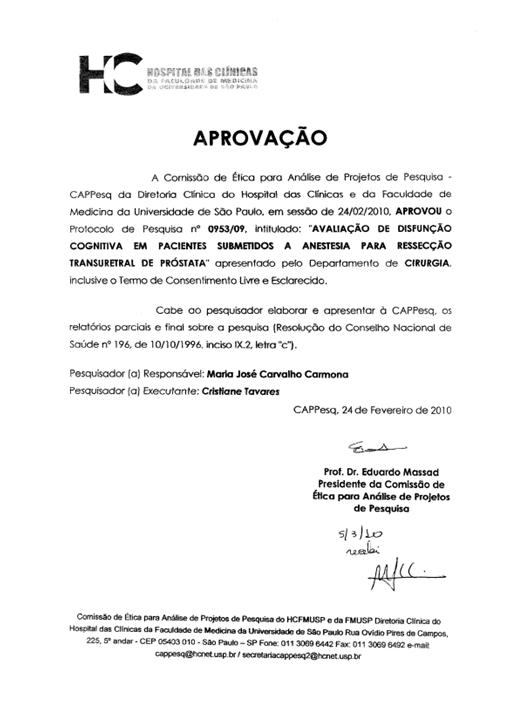


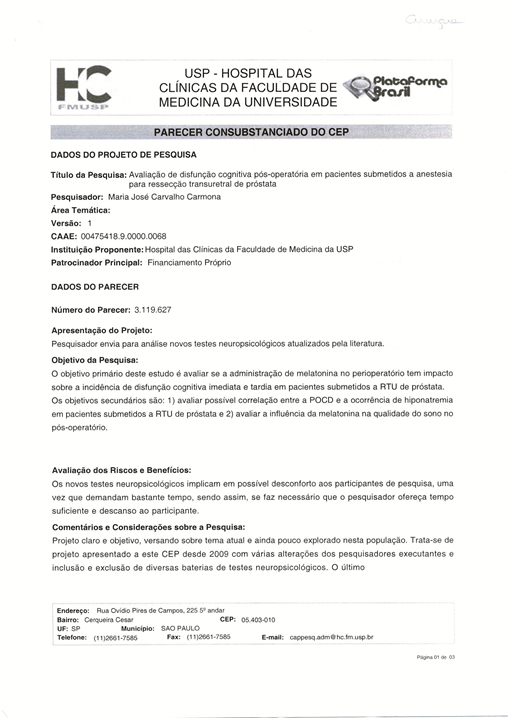


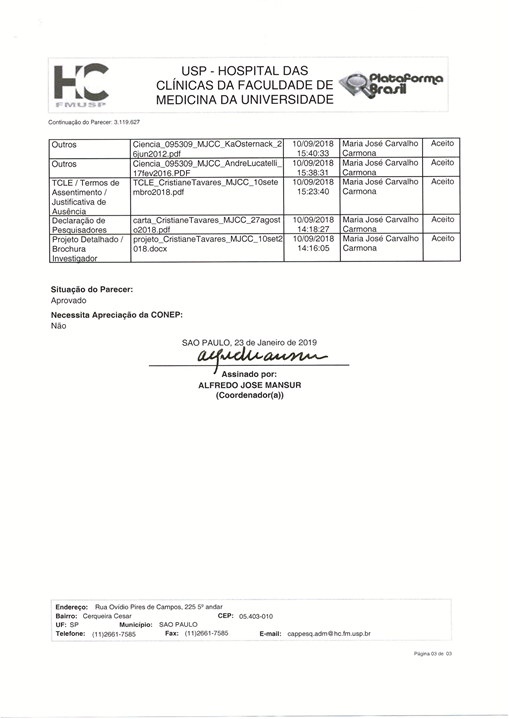

Supplement: Supplementary file 1 [file mmc1.docx]

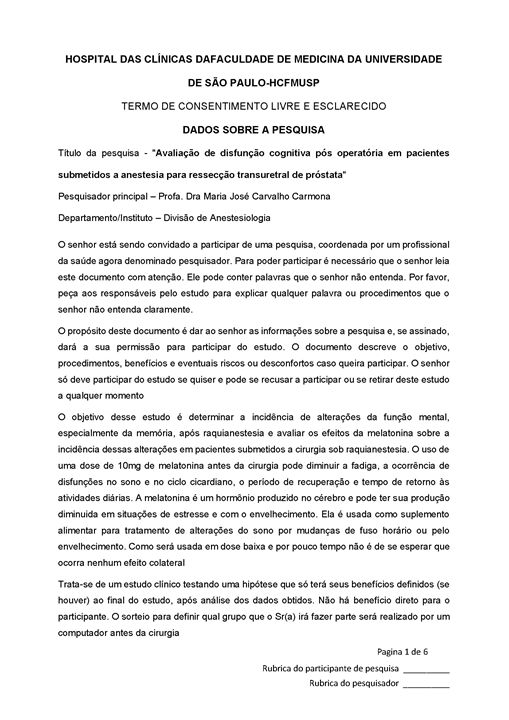


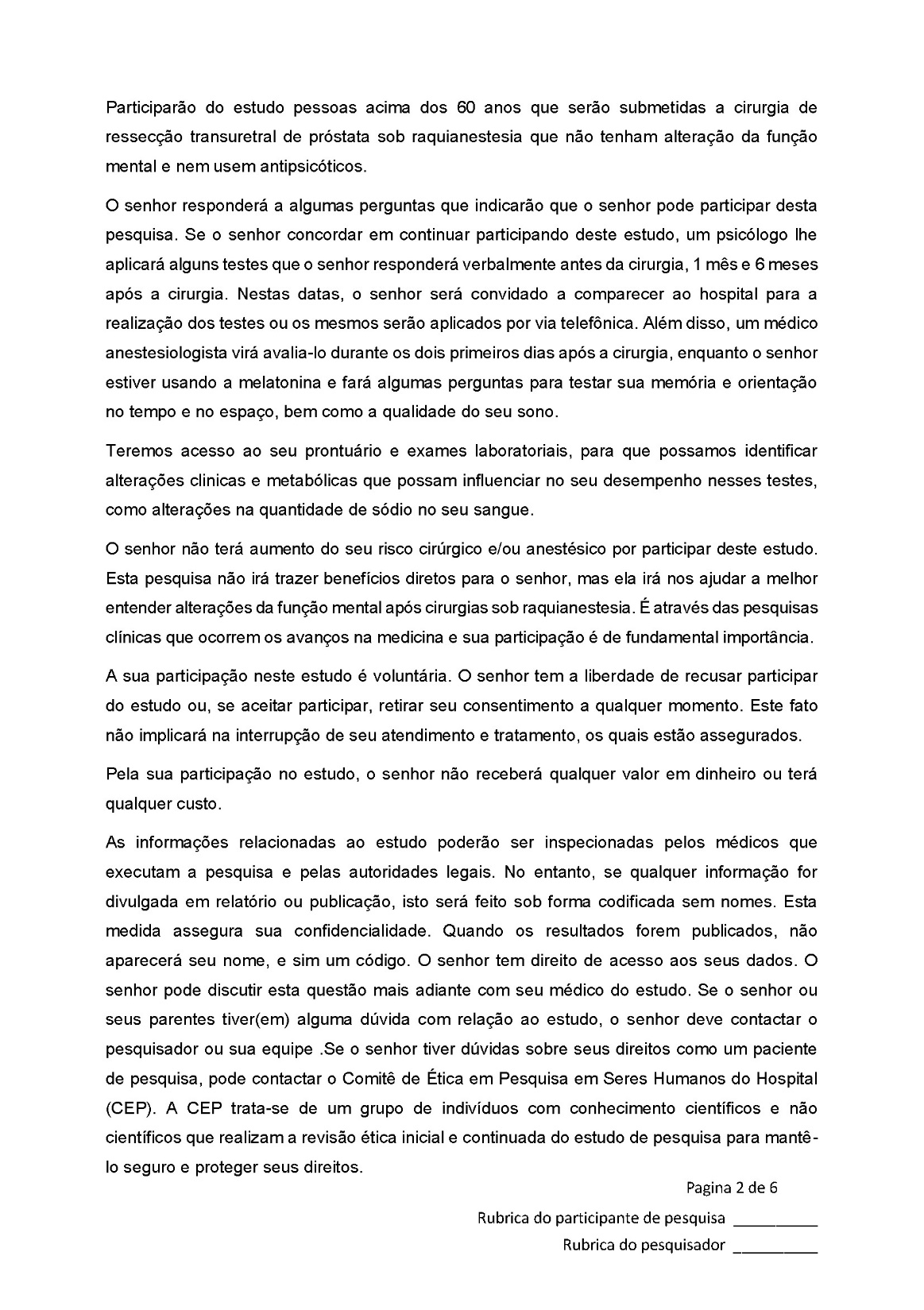


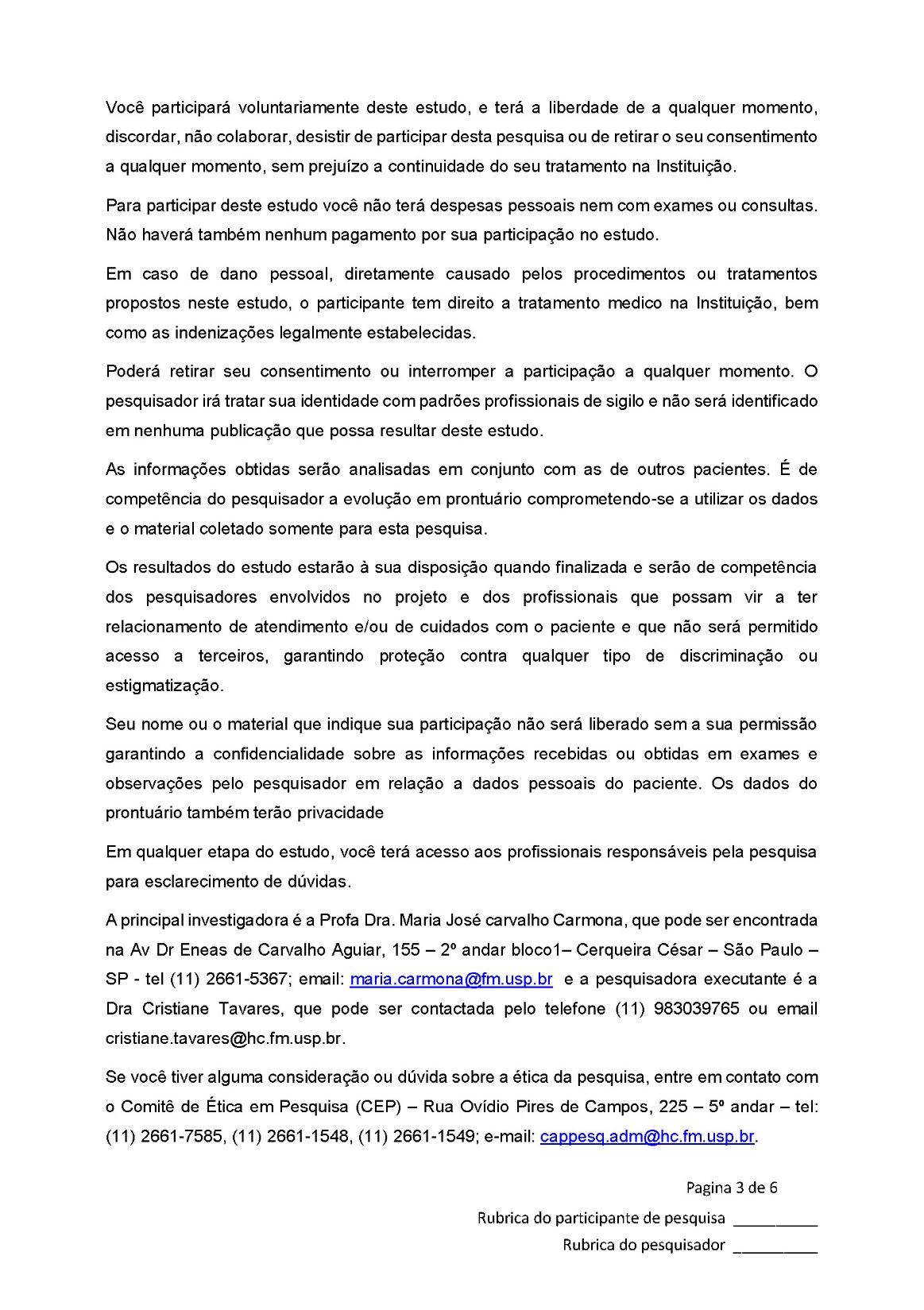


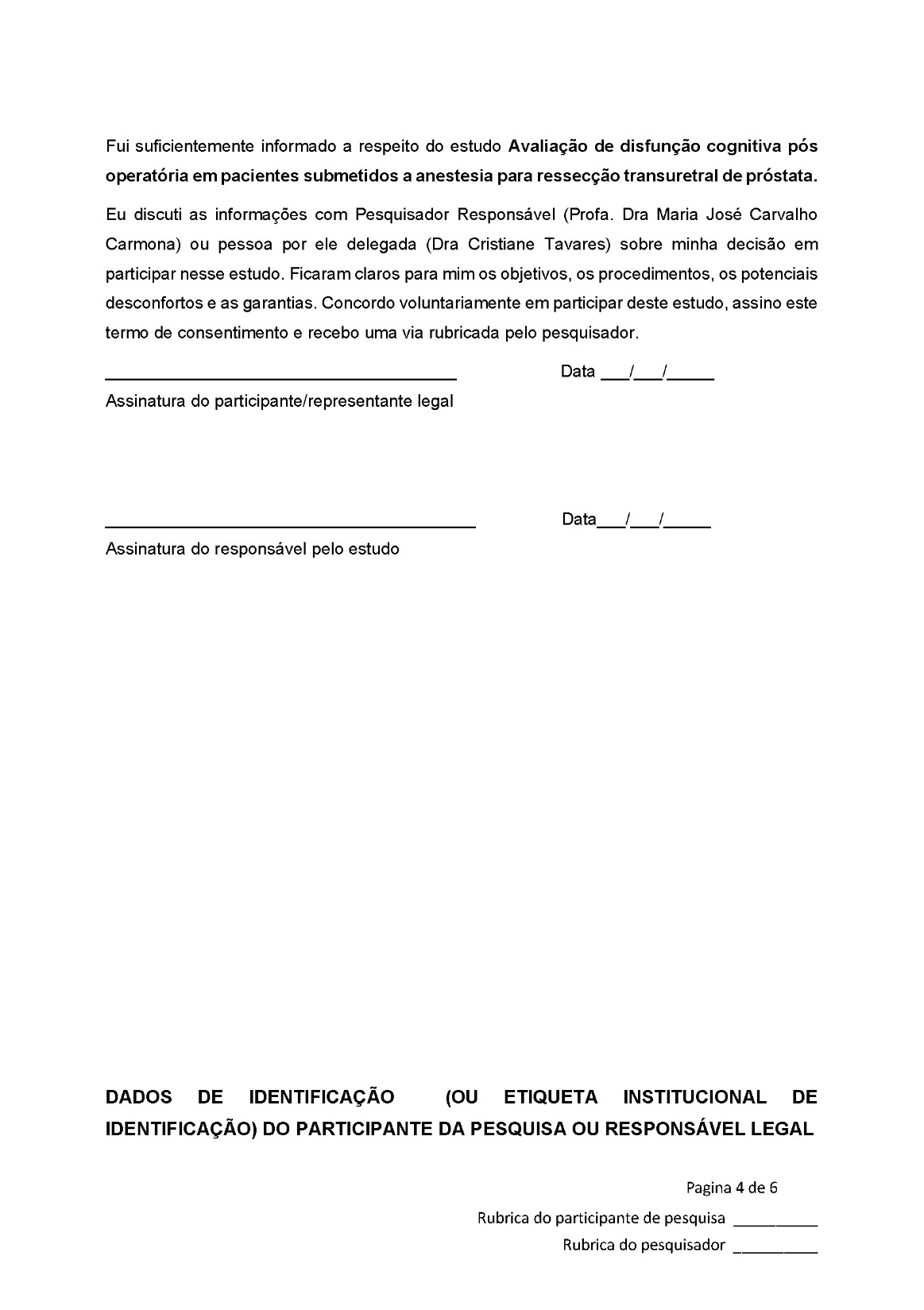

Supplement: Supplementary file 3 [file mmc3.docx]
