# Supplementary material for: Effect of melatonin on postoperative cognitive function in elderly patients submitted to transurethral resection of the prostate under spinal anesthesia
Source: Clinics (Sao Paulo). 2024 Dec 26;80:100562. doi: 10.1016/j.clinsp.2024.100562 (PMC11732585; doi:10.1016/j.clinsp.2024.100562)
Supplement: Supplementary file 2 [file mmc2.pdf]

ClinicalTrials.gov Search Results 01/12/2020

	Title	Status	Study Results	Conditions	Interventions	Locations
1	Use of Melatonin for Preventing POCD in Transurethral Prostate Resection Under Spinal Anesthesia	Recruiting	No Results Available	•Melatonin<br>•Cognitive Dysfunction<br>•Postoperative Complications<br>•Prostate Hyperplasia	•Dietary Supplement: melatonin<br>•Drug: Placebo oral tablet	•Hospital das Clinicas, São Paulo, SP, Brazil

U.S. National Library of Medicine | U.S. National Institutes of Health | U.S. Department of Health & Human Services
